# Supplementary material for: Light Quality-Dependent Regulation of Non-Photochemical Quenching in Tomato Plants
Source: Biology (Basel). 2021 Jul 28;10(8):721. doi: 10.3390/biology10080721 (PMC8389287; doi:10.3390/biology10080721)
Supplement: Supplementary file 1 [file biology-10-00721-s001.zip › biology-1283447-supplementary.pdf]

Supplementary Materials

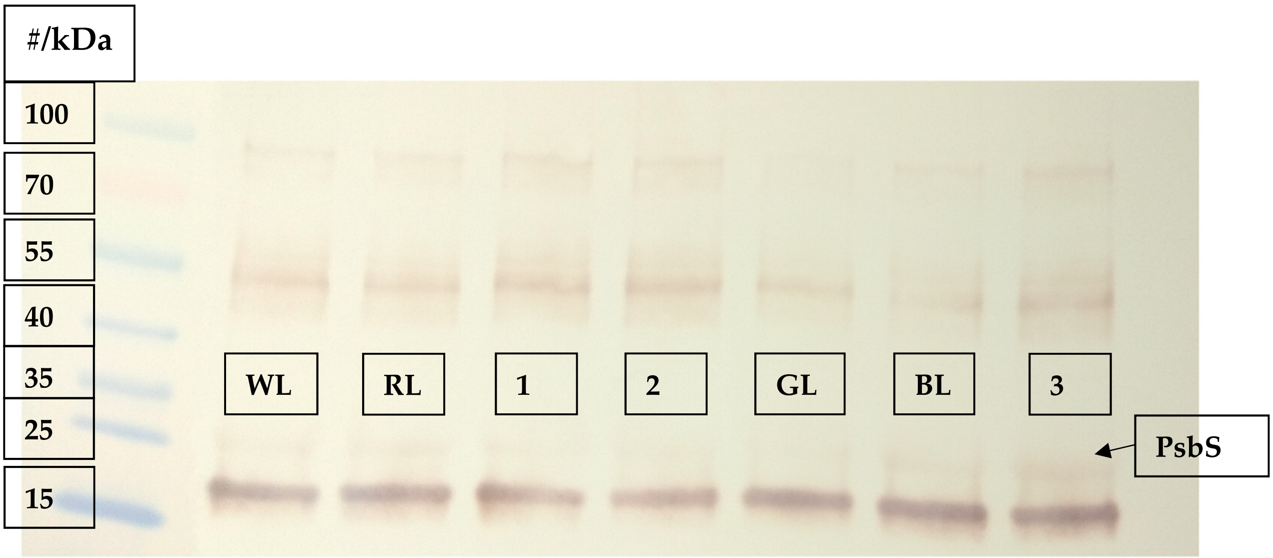

Figure S1. Western blot membrane of PsbS (~22 kDa) protein detected with anti-PsbS (AS09 533; 1:1000; Agrisera, Vännäs, Sweden) antibody. Gel-separated proteins were transferred to nitrocellulose membranes (0.2 µm pore size; Bio-Rad, Hercules, USA) by semi-dry electroblotting (1.5 mA per cm^2, 20 min). Membranes, incubated with a horseradish peroxidase-conjugated secondary antibody (AS09 602; 1:5000–1:10000; Agrisera), were developed with Pierce™ DAB Substrate Kit (Thermo Fisher Scientific). #Weight marker (molecular weight in kDa): Thermo Scientific™/PageRuler™ Prestained Protein Ladder, 10 to 180 kDa; catalogue number: 26616. Blot images, prior to the densitometry readings, were converted to grayscale with ImageJ (ImageJ v.1.49, National Institutes of Health, Maryland, USA) as follows: Image -> Type –> 8 bit, next: Image -> Adjust–> Brightness/Contrast –> Auto. WL – the R:G:B = 1:1:1 (referred to as the WL—white light) at 120 µmol m^{-2} s^{-1}; RL, GL or BL – monochromatic red (R), green (G) or blue (B) light (L) at 80 µmol m^{-2} s^{-1}, respectively. 1, 2 and 3 – additional treatments.

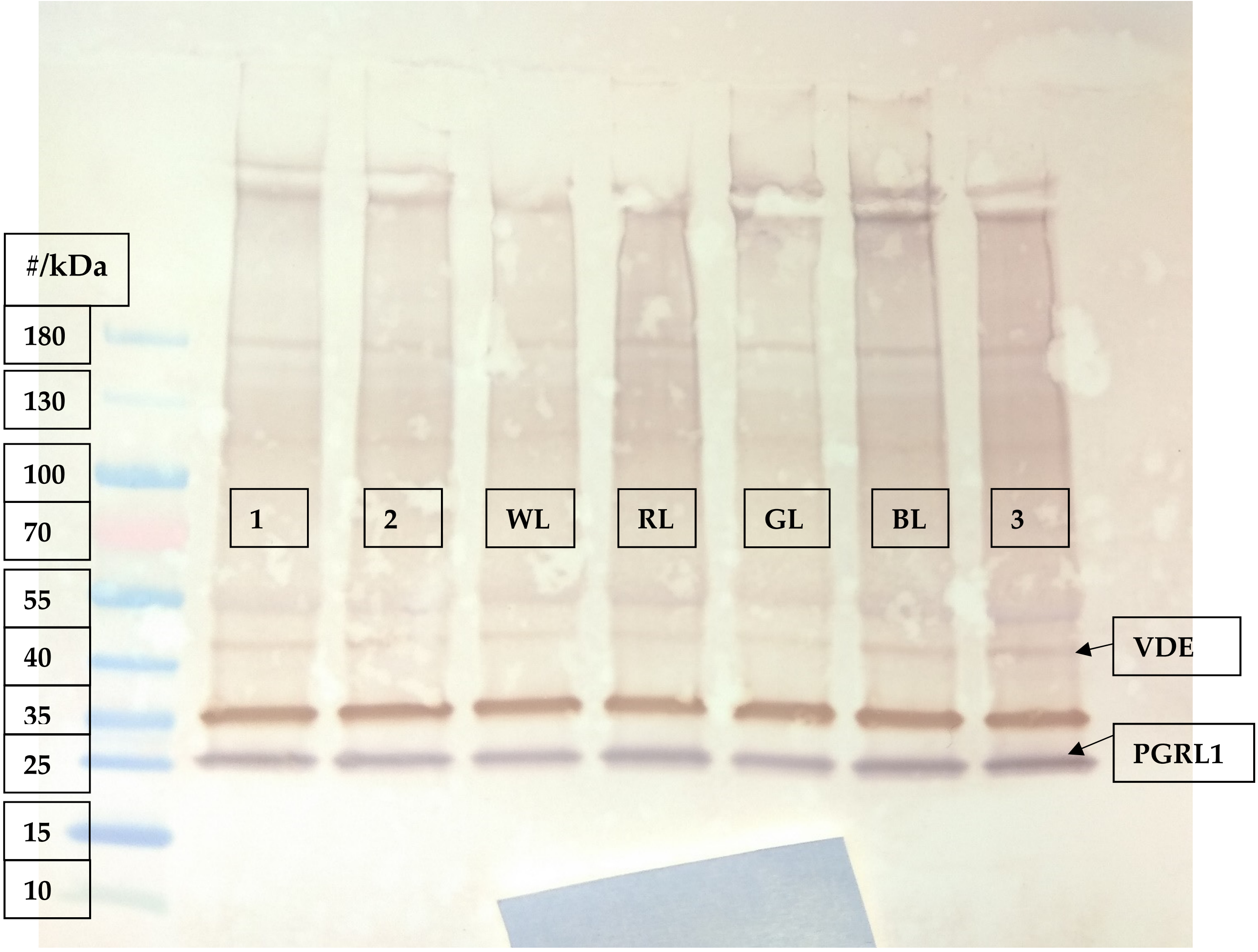

Figure S2. Western blot membrane of VDE (~40 kDa) and PGRL1 (~29 kDa) protein detected with anti-VDE (AS15 3091; 1:1000; Agrisera) and anti-PGRL1 (AS10 725/AS19 4311; 1:1000; Agrisera) antibodies, respectively. Gel-separated proteins were transferred to nitrocellulose membranes (0.45 µm pore size; Bio-Rad) by semi-dry electroblotting (1.5 mA per cm^2, 20 min). Membranes, incubated with a horseradish peroxidase-conjugated secondary antibody (AS09 602; 1:5000–1:10000; Agrisera), were developed with Pierce™ DAB Substrate Kit (Thermo Fisher Scientific). #Weight marker (molecular weight in kDa): Thermo Scientific™/PageRuler™ Prestained Protein Ladder, 10 to 180 kDa; catalogue number: 26616. Blot images, prior to the densitometry readings, were converted to grayscale with ImageJ (ImageJ v.1.49, National Institutes of Health) as follows: Image -> Type –> 8 bit, next: Image -> Adjust–> Brightness/Contrast –> Auto. WL – the R:G:B = 1:1:1 (referred to as the WL—white light) at 120 µmol m^{-2} s^{-1}; RL, GL or BL – monochromatic red (R), green (G) or blue (B) light (L) at 80 µmol m^{-2} s^{-1}, respectively. 1, 2 and 3 – additional treatments.

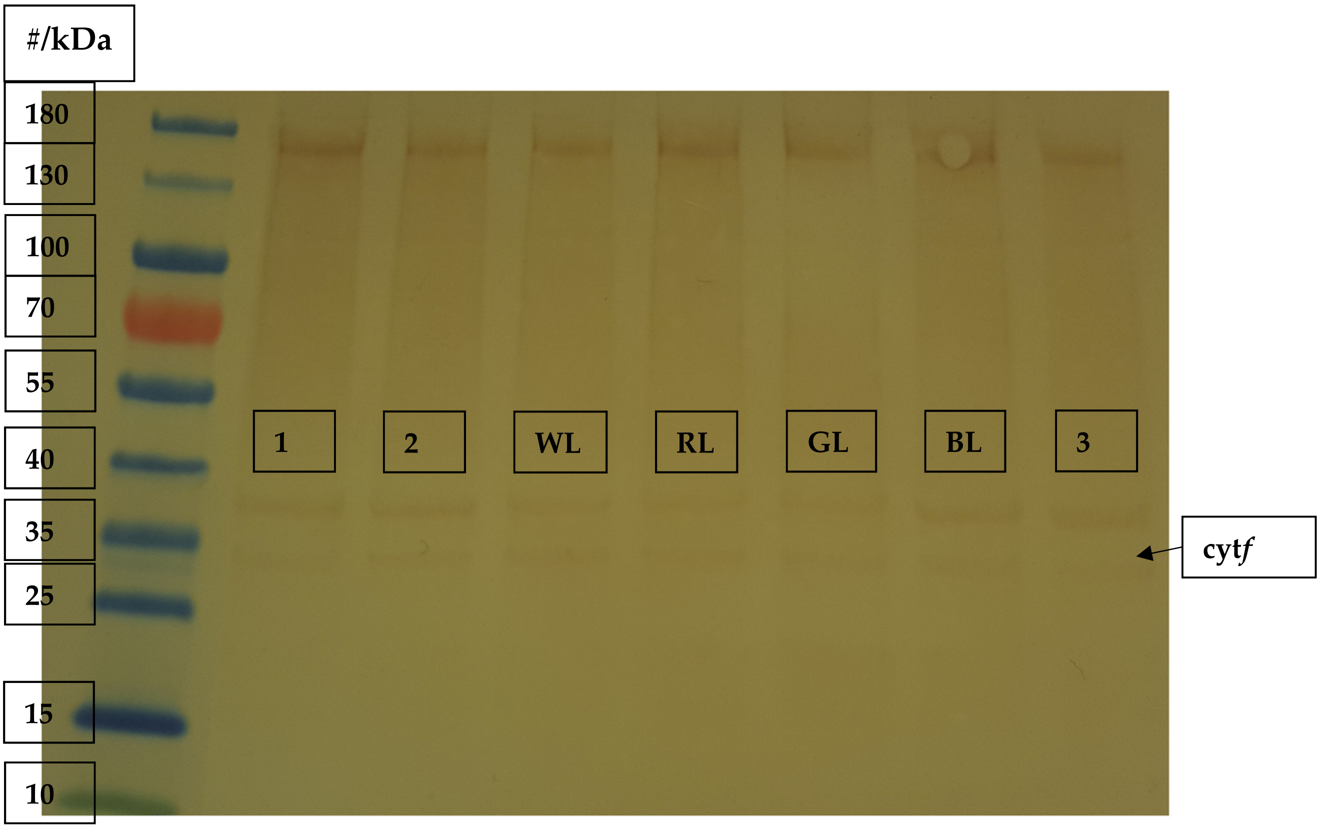

Figure S3. Western blot membrane of cyt*f* (~32 kDa) protein detected with anti-cyt*f* (AS08 306; 1:5000; Agrisera) antibody. Gel-separated proteins were transferred to nitrocellulose membranes (0.2 µm pore size; Bio-Rad) by semi-dry electroblotting (1.5 mA per cm^2, 20 min). Membranes, incubated with a horseradish peroxidase-conjugated secondary antibody (AS09 602; 1:5000–1:10000; Agrisera), were developed with Pierce™ DAB Substrate Kit (Thermo Fisher Scientific). #Weight marker (molecular weight in kDa): Thermo Scientific™/PageRuler™ Prestained Protein Ladder, 10 to 180 kDa; catalogue number: 26616. Blot images, prior to the densitometry readings, were converted to grayscale with ImageJ (ImageJ v.1.49, National Institutes of Health) as follows: Image -> Type –> 8 bit, next: Image -> Adjust–> Brightness/Contrast –> Auto. WL – the R:G:B = 1:1:1 (referred to as the WL—white light) at 120 µmol m^{-2} s^{-1}; RL, GL or BL – monochromatic red (R), green (G) or blue (B) light (L) at 80 µmol m^{-2} s^{-1}, respectively. 1, 2 and 3 – additional treatments.

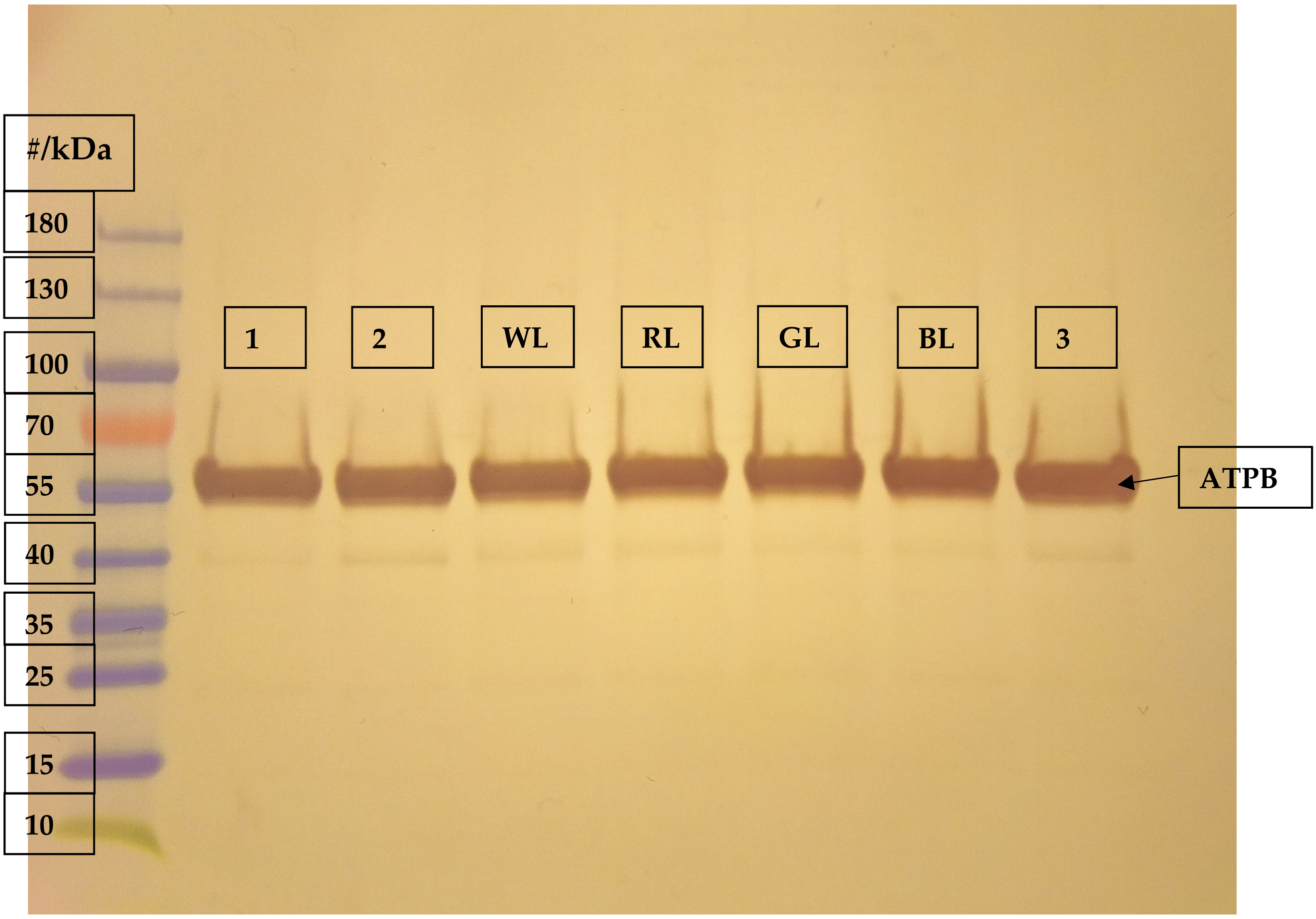

Figure S4. Western blot membrane of ATPB (~55 kDa) protein (loading control) detected with anti-ATPB (beta subunit of ATP synthase; AS05 085; 1:5000; Agrisera) antibody. Gel-separated proteins were transferred to nitrocellulose membranes (0.45 μm pore size; Bio-Rad) by semi-dry electroblotting (1.5 mA per cm^2, 20 min). Membranes, incubated with a horseradish peroxidase-conjugated secondary antibody (AS09 602; 1:5000–1:10000; Agrisera), were developed with Pierce™ DAB Substrate Kit (Thermo Fisher Scientific). #Weight marker (molecular weight in kDa): Thermo Scientific™/PageRuler™ Prestained Protein Ladder, 10 to 180 kDa; catalogue number: 26616. Blot images, prior to the densitometry readings, were converted to grayscale with ImageJ (ImageJ v.1.49, National Institutes of Health) as follows: Image -> Type –> 8 bit, next: Image -> Adjust–> Brightness/Contrast –> Auto. WL – the R:G:B = 1:1:1 (referred to as the WL—white light) at 120 μmol m^{-2} s^{-1}; RL, GL or BL – monochromatic red (R), green (G) or blue (B) light (L) at 80 μmol m^{-2} s^{-1}, respectively. 1, 2 and 3 – additional treatments.
